# Are arch width measurements from Invisalign arch width tables reliable?

**DOI:** 10.1007/s00784-026-06904-w

**Published:** 2026-05-13

**Authors:** Cristina de-la-Rosa-Gay, Sofia Valmaseda-de-la-Rosa, Andrea Hernández-Mangas, Octavi Camps-Font, Eduard Valmaseda-Castellón, Rui Figueiredo

**Affiliations:** 1 Department of Dentistry, School of Medicine and Health Sciences, Campus Bellvitge, Universitat de Barcelona, Carrer Feixa Llarga s/n. L’Hospitalet de Llobregat, Barcelona, 08907 Spain; 2 Group of Dental and Maxillofacial Pathology and Therapeutics, IDIBELL Research Institute, Carrer Feixa Llarga s/n. L’Hospitalet de Llobregat 08907, Barcelona, 08907 Spain; 3 Private dental practice, Espai Dental La Garriga, Carrer de Samalús 3, La Garriga, Barcelona, 08530 Spain

**Keywords:** Clear aligner therapy, Invisalign, Digital models, Agreement, Expansion, Validation

## Abstract

**Introduction:**

Despite being widely used for treatment planning, the accuracy of Invisalign arch width tables has not been independently assessed. The objective of this study was to assess whether the predicted and observed arch width changes calculated from Invisalign tables are consistent with measurements obtained from STL models.

**Methods:**

Thirty-five adults treated with Invisalign aligners were retrospectively selected. Arch width at the maxillary and mandibular canines, premolars, and first molars was measured on digital models (pretreatment, prediction and first-refinement) using Geomagic Control X. Predicted and observed expansions (difference between predicted or post-treatment and pretreatment arch widths), and their discrepancy, were compared with the corresponding values calculated from the ClinCheck arch width tables. Three references were selected: (1) the projection of the long axis of the tooth on the occlusal surface, (2) the buccal/mesiobuccal cusps, and (3) the most lingual point of the gingival margin. Normality was assessed with the Shapiro-Wilk test. Agreement was evaluated using Bland-Altman analysis with mixed-effects models to account for clustering of repeated measurements.

**Results:**

840 arch widths were analyzed (35 patients, 4 tooth pairs, 2 jaws, and 3 time points). Non-normality of inter-method differences was observed in predicted expansion (gingival and occlusal references) and in discrepancy (cusp reference) (*p* < 0.05). Non-parametric Bland-Altman analysis showed high agreement between Geomagic and ClinCheck measurements for predicted expansion, observed expansion, and discrepancy, with bias values ranging from − 0.49 to 0.2 mm.

**Conclusions:**

ClinCheck arch width tables showed strong agreement with independent metrological assessment, particularly with occlusal reference points [bias: 0.00 mm; limits of agreement: −0.80 to 1.01 mm].

**Clinical relevance:**

ClinCheck arch width tables have been validated with an independent metrological assessment (Geomagic Control X). Predicted expansion, observed expansion, and discrepancy derived from arch width tables agreed with independent measurements using virtual casts.

## Objectives

Clear aligner therapy (CAT) has become a widely adopted approach for managing malocclusions; [1] Invisalign is one of the most prescribed systems worldwide [2–5]. Moreover, CAT is attracting increasing academic interest in its performance, digital workflow, and clinical outcomes [6]. A key advantage of CAT lies in its integration of three-dimensional digital technology for both treatment planning and aligner fabrication. Proprietary platforms such as Invisalign ClinCheck (Align Technology, San Jose, CA, USA) help clinicians virtually plan tooth movements and visualize the predicted treatment outcome. Additionally, ClinCheck provides data on initial and predicted overjet, overbite, and arch width.

Despite the widespread use of ClinCheck, limited data exists regarding the accuracy of measurements, and Align Technology has not provided data regarding their validity [7]. Recent studies have raised concerns about the reliability of other ClinCheck measurements, such as angles and mesiodistal tooth size [8, 9]. Although a recent validation study confirmed that the overjet and overbite changes predicted by Invisalign’s ClinCheck digital treatment-planning system are consistent with measurements obtained using independent metrology software [10], the validity of transverse dimension measurements in ClinCheck tables has not yet been independently assessed.

Transverse arch control remains a key objective in orthodontic treatment, and numerous studies on the predictability of CAT have specifically addressed it [7, 11–16]. Reliable assessment of arch width measurements reported by ClinCheck is essential for both clinicians and researchers. Validated data could streamline research workflows by reducing the need for time-intensive metrological analyses and enhance clinical confidence in using ClinCheck data for treatment planning.

To evaluate treatment changes, orthodontic research relies on 3D metrology software such as software calipers [8, 17], MeshLab [7], or Meazure [12, 18]. Geomagic Control X (Hexagon AB, Stockholm, Sweden) is recognized for its precision and has been used extensively to assess changes in occlusal relationships and tooth positions in CAT [10, 12, 17, 19–25].

This study aimed to determine whether the predicted and the observed dental arch expansion provided by Align Technology’s ClinCheck are consistent with measurements of STL models using Geomagic Control X metrology software.

## Materials and methods

### Study design and ethical considerations

A retrospective cohort of patients treated with SmartTrack aligners (Align Technology, San José, CA, USA) between November 2017 and September 2025 was selected. All treatments were performed by a single orthodontist (CDLRG) in a private dental clinic in the Barcelona metropolitan area (Spain).

All procedures were performed in compliance with relevant laws and institutional guidelines and were approved by the Ethics Committee of the Dental Hospital University of Barcelona (approval date: 22/7/2025; protocol number: 49/2025). Reporting adhered to the STROBE guidelines [26] and was conducted in accordance with the principles of the Declaration of Helsinki [27]. Informed consent was waived due to the retrospective and anonymized nature of the data collection.

### Participant selection

Patients were consecutively screened from the clinic’s records starting in November 2017 until the predefined sample size was reached. The first 35 consecutive patients fulfilling all criteria were included in the final analysis (126 were screened and 91 were not eligible).

### Inclusion and exclusion criteria

Participants met the following inclusion criteria: adults (aged 18 years or older), completion of orthodontic treatment with Invisalign SmartTrack aligners without dental extractions, and treatment involving both maxillary and mandibular arches. Exclusion criteria included absence of any central incisor, canine, premolar, first or second molar, use of auxiliaries beyond attachments or Class II/III elastics, poor aligner compliance or frequent misfits during appointments, and absence of a first refinement phase.

### Variables and statistical analysis

**Fig. 1:**
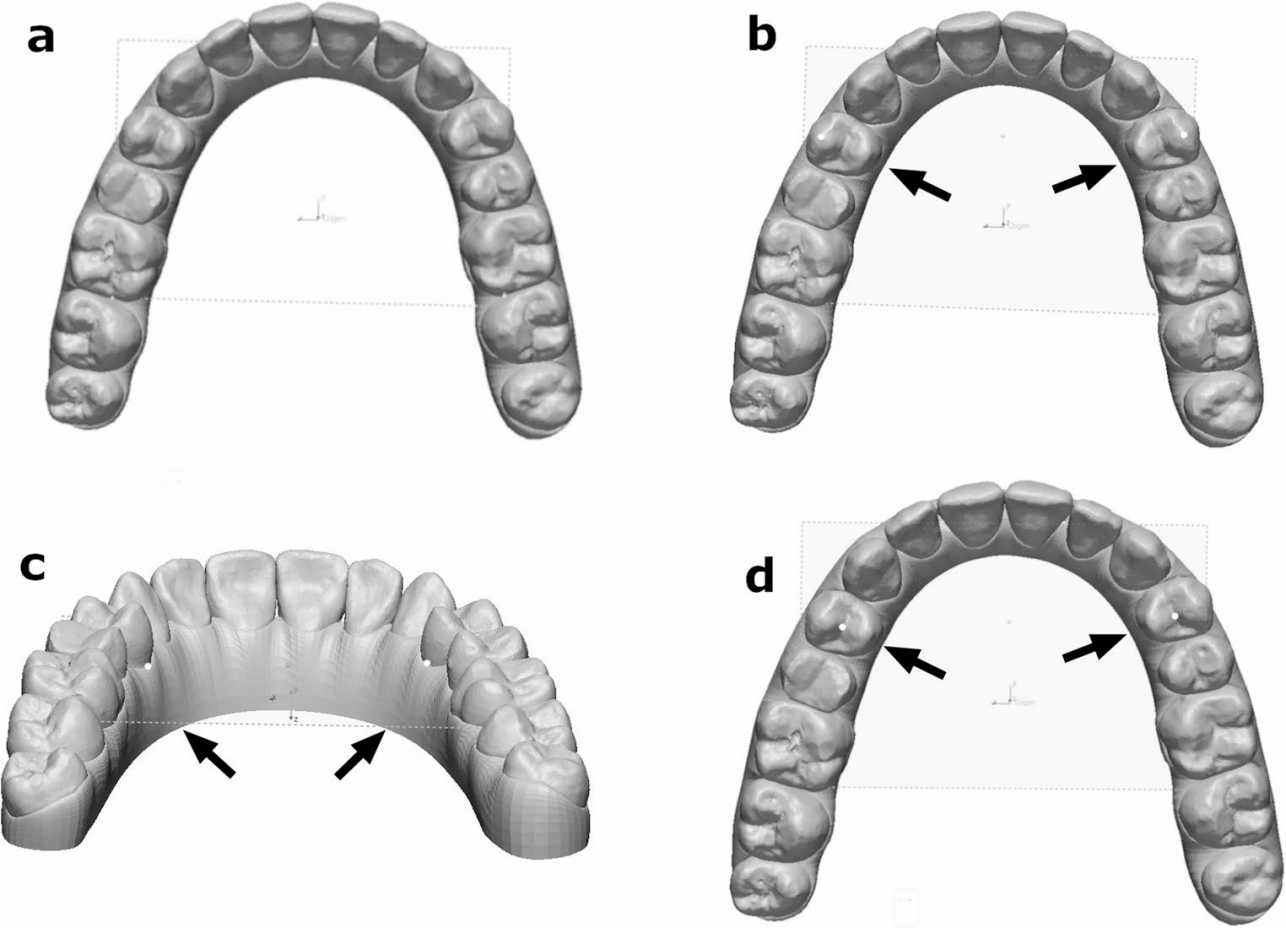
(**a**) The reference was established using a horizontal plane passing through the interproximal papilla between the maxillary first and second molars and the center of the superior margin of the incisive papilla. Arch width measurements were made along the x-plane. (**b**) Cusp reference points in buccal cusps of the first premolars (light dots). (**c**) Gingival reference points of the first premolars (light dots). (**d**) Occlusal reference points at the projection of the long anatomical axis of the tooth on the occlusal surface of the first premolars (light dots). All measurements were made in millimeters

Initial, predicted, and observed dental arch widths between the maxillary and mandibular canines, first and second premolars, and first molars were retrieved from the ClinCheck arch width tables. The initial and predicted dental arch width originated from the first approved ClinCheck plan, while the observed dental arch width was obtained from the initial model of the first refinement stage. To ensure blinding, a researcher unaware of these values (SVDLR) measured dental arch widths using Geomagic Control X^®^ version 2023.3.0 (Hexagon AB, Stockholm, Sweden). After importing STL files, following the protocol established in a previous validation study [10], a horizontal reference plane was drawn connecting three points: the palatal interproximal papillae between the maxillary first and second molars, and the midpoint of the superior margin of the papilla between both central incisors (Fig. 1). The initial, predicted, and observed digital models (STL files) were aligned to this reference plane [21, 28]. This step ensured that all models shared a consistent spatial orientation, allowing linear distances to be measured along comparable axes across time points.

Dental arch width was measured as the horizontal linear distance between canines, first and second premolars, and first molars in each dental arch. Despite reports indicating that ClinCheck arch width tables use the projection of the long axis of the tooth on the occlusal surface, Align Technology has not released the computational algorithm [7]. Therefore, we included three different reference points—occlusal, cusp, and gingival—each of which has been used in previous studies to assess whether agreement with ClinCheck values was consistent across different measurement levels [7, 17, 18]. The three reference points used were (Fig. 1):

- Occlusal: the point on the occlusal surface intersecting the long anatomical axis of each tooth [7].
- Cusp: cusps of the canines, buccal cusps of the first and second premolars, and mesio-buccal cusps of the first molar [7, 17, 18].
- Gingival: the most lingual point of the gingival margin of each tooth [7, 17, 18].

Potential sources of measurement variability, such as dental wear, irregular gingival margins, and minor differences in STL alignment, were not controlled for in this study. Nevertheless, repeated measurements performed after at least 15 days in 10 randomly selected cases yielded very high intraclass correlation coefficients (ICCs; 0.989–0.997), indicating excellent test–retest reliability despite these potential sources of variability.

As noted in a previous validation study, the reference points and centers of rotation used by ClinCheck cannot be reproduced with external metrology software [7, 10]. Consequently, direct comparisons of pretreatment, predicted, and pre-refinement arch widths were not feasible. To overcome this, three variables were calculated: predicted expansion (predicted dental arch width minus pretreatment dental arch width), observed expansion (post-treatment dental arch width minus pretreatment dental arch width), and the discrepancy between predicted and observed expansion (observed expansion minus predicted expansion). Normality of the inter-method differences between Geomagic and ClinCheck measurements was assessed using Shapiro-Wilk tests. Agreement between Geomagic and ClinCheck measurements was assessed using Bland–Altman analysis applied to paired observations. Because each patient contributed multiple non-independent measurements, clustering was addressed using mixed-effects models with patient-specific random intercepts. The magnitude of within-patient clustering across the 24 repeated measurements per participant was quantified using the ICC, which ranged from 0.37 to 0.46 across the three expansion outcomes. Bias (mean difference) and 95% limits of agreement (LoA) were estimated using variance components from the Mixed-effects Bland–Altman model: (i) population-level LoA based on the total variance (between-patient and within-patient), and (ii) within-patient LoA based on the within-patient variance only, reflecting agreement for repeated measurements within the same participant. Analyses were performed in R (version 4.0; R Core Team, Vienna, Austria), using RStudio as the development environment.

No missing data was present due to inclusion criteria ensuring complete dentitions and availability of all STL models; no imputation was required.

### Sample size calculation

A previous validation of Invisalign ClinCheck overjet and overbite tables included 76 patients and 2 observations per patient (pretreatment and predicted) for each outcome [10]. In the present study, we included 35 patients, each contributing three time points (pretreatment, predicted, and before the first refinement) and 8 transverse arch width values (4 maxillary and 4 mandibular), resulting in 24 repeated measurements per patient (840 paired measurements in total). Because the precision of Bland–Altman limits of agreement is driven primarily by the number of independent subjects rather than by the number of repeated measurements, our a priori target was to enroll a minimum of 30 patients, consistent with prior validation work in this field [10]. To quantify the impact of within-patient clustering, we estimated the ICC across the 24 repeated measurements per participant from the study data (ICC = 0.37 for discrepancy, 0.45 for observed expansion, and 0.46 for predicted expansion), which corresponds to a design effect of 9.6 to 11.7 and an effective sample size of approximately 72 to 88 independent paired observations for estimating the mean bias at the measurement level. Importantly, all agreement estimates were obtained using Mixed-effects Bland–Altman models with patient-level random intercepts (thereby treating the patient as the primary sampling unit), and complementary non-parametric LoA were reported to accommodate the non-normality observed in some distributions.

## Results

Of the patients initially screened, 35 met the inclusion criteria and were analyzed, contributing a total of 280 paired measurements across occlusal, cusp, and gingival reference points at each time point. Because measurements were repeated at three time points (pretreatment, predicted, and before the first refinement), a total of 840 paired measurements were obtained.

The Shapiro-Wilk test applied to the differences between Geomagic and ClinCheck measurements indicated that some distributions (predicted expansion in the gingival and the occlusal regions, and discrepancy in the cusp region) deviated from normality (*p* < 0.05) (Table 1). These deviations suggest that predicted expansion values, particularly when using gingival references, are more prone to non-normal distributions, which might reflect a greater measurement variability. In contrast, observed expansion remained normally distributed across all references, and discrepancy was generally robust, with only minor deviation at cusp references. This pattern supports the relative stability of occlusal and cusp references compared with gingival margins.

**Table 1:** Shapiro-Wilk test results for inter-method differences (Geomagic vs ClinCheck) across measurement references

Dataset	Variable	Statistic (W)	*P*-value
Occlusal	Predicted expansion	0.98930	0.0384
	Observed expansion	0.99209	0.1442
	Discrepancy	0.99158	0.1133
Cusp	Predicted expansion	0.99132	0.1004
	Observed expansion	0.99680	0.8541
	Discrepancy	0.98898	0.0329
Gingival	Predicted expansion	0.98702	0.0131
	Observed expansion	0.99341	0.2624
	Discrepancy	0.99434	0.3892

W indicates the test statistic for the Shapiro-Wilk test; *p*-value is the significance level for rejecting the null hypothesis (normality). Predicted expansion rejected the normality assumption in gingival and occlusal regions (*p* < 0.05). Observed expansion was normal in all cases and discrepancy was non-normal only in the cusp region (*p* < 0.05)

**Fig. 2:**
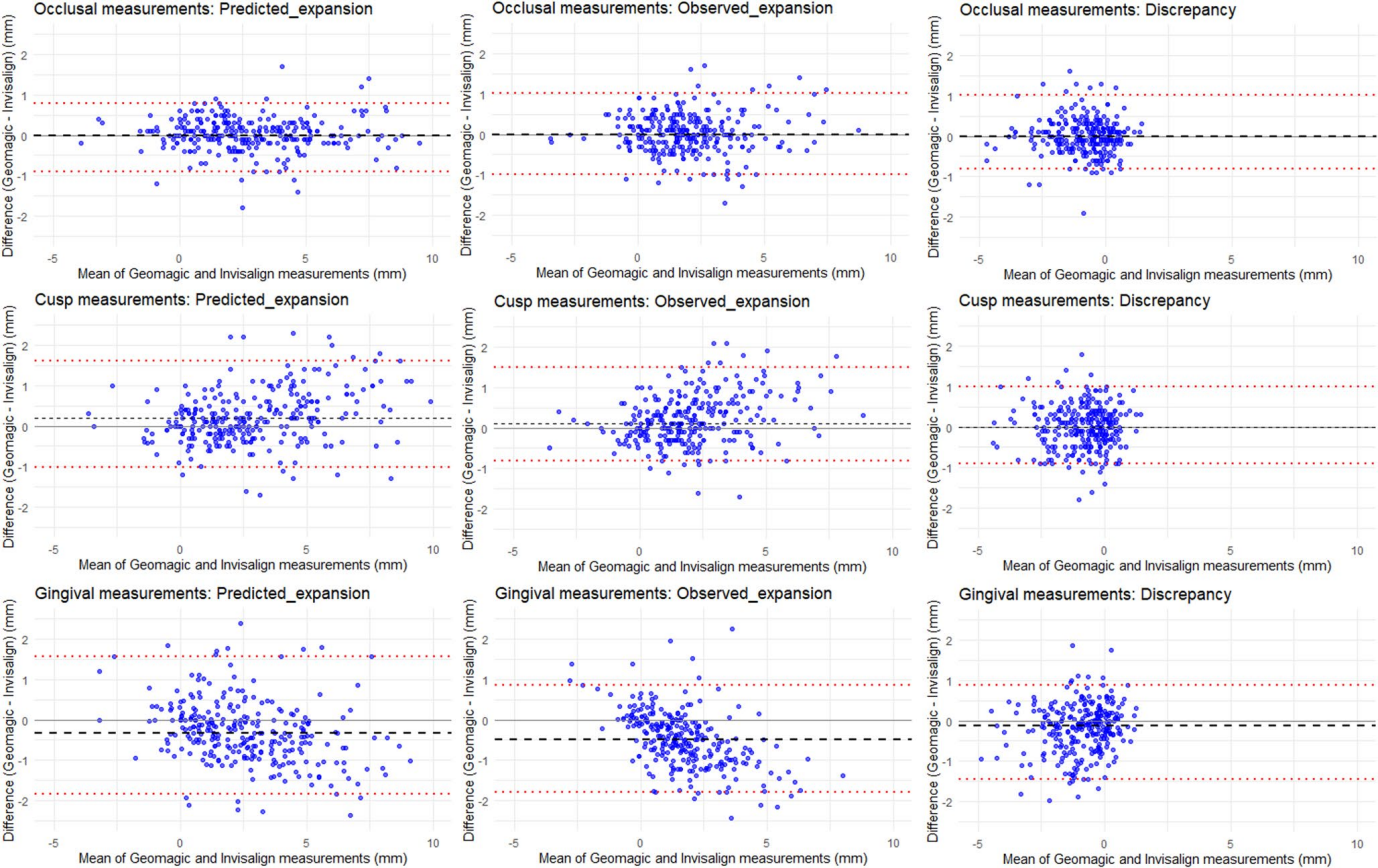
Non-Parametric Bland-Altman agreement plots between the measured values (Geomagic) and those provided by the ClinCheck tables. Graphs display agreement for the different expansion variables: i.e. predicted expansion (first column), observed expansion (second column), and discrepancy (third column). The reference points were the projection of the long anatomical axis of the tooth on the occlusal surface (first row), the cusps (second row) and the gingival margin (third row). The x-axis represents the mean of the two measurements (in mm), and the y-axis represents the difference between Geomagic and Invisalign measurements (in mm). The dashed black line represents the median difference (bias), and the dashed red lines indicate the limits of agreement (LoA) (2.5th and 97.5th percentiles). Plots are shown at the level of individual measurements, prior to accounting for within-patient clustering. The limits of agreement represent the statistical distribution of differences, not predefined clinical thresholds; ranges within approximately ± 1 mm for occlusal and cusp references and up to ± 1.8 mm for gingival references might be considered acceptable in orthodontic practice

Therefore, non-parametric statistics were used, including median values, interquartile ranges, and the 2.5th and 97.5th percentiles to estimate LoA. In addition, 95% confidence intervals for these limits were obtained using bootstrap resampling (2,000 iterations) (Table 2). Agreement between Geomagic and ClinCheck measurements was assessed for predicted expansion, observed expansion, and discrepancy across the three references using both non-parametric (Fig. 2) and Mixed-effects Bland-Altman analyses with patient random intercepts to account for clustering of repeated measurements.

**Table 2:** Non-parametric Bland-Altman agreement statistics between Geomagic and ClinCheck measurements, stratified by reference point and variable

Reference	Variable	Median (bias)	Lower LoA (95% CI)	Upper LoA (95% CI)	RoA	IQR
Occlusal	Predicted expansion	0.00	–0.90 (–1.10 to –0.70)	0.80 (0.60 to 0.91)	1.70	0.50
	Observed expansion	0.00	–1.00 (–1.11 to –0.82)	1.01 (0.81 to 1.21)	2.01	0.60
	Discrepancy	0.00	–0.80 (–0.91 to –0.62)	1.01 (0.70 to 1.30)	1.81	0.40
Cusp	Predicted expansion	0.20	–1.01 (–1.30 to –0.70)	1.61 (1.40 to 2.20)	2.61	0.80
	Observed expansion	0.10	–0.80 (–1.01 to –0.63)	1.51 (1.30 to 1.80)	2.31	0.80
	Discrepancy	0.00	–0.91 (–1.11 to –0.90)	1.00 (0.90 to 1.20)	1.91	0.60
Gingival	Predicted expansion	–0.33	–1.84 (–2.11 to –1.44)	1.57 (1.07 to 1.77)	3.41	0.85
	Observed expansion	–0.49	–1.80 (–2.00 to –1.66)	0.86 (0.59 to 1.39)	2.66	0.91
	Discrepancy	–0.12	–1.45 (–1.68 to –1.32)	0.87 (0.61 to 1.09)	2.33	0.72

*LoA* indicates Limits of Agreement (non-parametric 2.5^th^ and 97.5^th^ percentiles); 95% CI, 95% Confidence Interval for the LoA estimated via bootstrapping; *RoA*, Range of Agreement; and *IQR*, Interquartile Range. All linear measurements are presented in millimeters (mm) and rounded to two decimal places. Mixed effects Bland-Altman analyses yielded similar estimates of bias and limits of agreement, while accounting for clustering of repeated measurements within patients (see text)

Across landmarks, occlusal and cusp references yielded LoA within approximately ± 1 mm, ranges generally considered acceptable for clinical monitoring of transverse changes. Gingival references showed wider limits (up to ± 1.8 mm), indicating greater variability and reduced reliability. Discrepancy values consistently produced narrower ranges and biases close to zero, supporting their use as a more stable metric (Table 2).

Mixed-effects Bland–Altman analysis of occlusal measurements showed minimal bias (0.03 mm) and 95% LoA between − 0.78 and + 0.84 mm (total variance) and − 0.74 to + 0.81 mm (within-patient variance), confirming strong agreement between ClinCheck and Geomagic values. For cusp measurements, small positive biases were observed (0.21 mm for predicted and observed expansion), with wider limits (approximately − 1.0 to + 1.5 mm). Gingival measurements showed small negative biases (− 0.30 mm for predicted expansion, − 0.48 mm for observed expansion, and − 0.18 mm for discrepancy), with limits up to ± 1.8 mm.

Overall, the observed LoA ( ≤ ± 1 mm for occlusal and cusp, ≤ ±1.8 mm for gingival) indicated that differences between ClinCheck and Geomagic are small relative to clinically relevant thresholds for arch width changes in orthodontic practice.

## Discussion

The occlusal reference, which is reportedly used by Align Technology in the arch width table [7], exhibited the highest concordance and precision across all variables. Conceptually, occlusal points can represent more biomechanically stable landmarks than cusp or gingival references, as they are less affected by anatomical variability, wear, or soft-tissue irregularities and changes. This stability likely explains the tighter agreement observed in our analyses and supports the occlusal points as a reliable reference for digital model validation.

Measurements based on cusp references also showed high concordance, closely mirroring the occlusal landmarks. While cusp tips are susceptible to attrition or flattening over time, their distinct anatomical nature makes them relatively easy to identify or extrapolate, which likely explains their consistent performance [29], even if minor positive biases are observed.

In contrast, measurements with the gingival reference showed the poorest agreement among the three references. This is not unexpected, as gingival contour or thickness may change with time, and are more difficult to segment. Such factors introduce greater measurement error and explain the observed wider LoA. While the average differences remained small and of limited clinical significance, this broader variability indicates that individual measurements may deviate more substantially. Clinicians should therefore interpret gingival-based expansion values with caution and rely preferentially on occlusal or cusp references, which provide more stable and reproducible reference points. These differences highlight the importance of carefully selecting anatomical references when evaluating digital orthodontic tools.

In contrast to correlation coefficients and t-tests, the Bland-Altman methodology accurately quantifies the systematic bias and compares precision of expansion measurements [10, 30, 31]. This analysis illustrates the agreement of dental arch expansion calculated from arch width measurements with Geomagic and Invisalign arch width tables yet simultaneously demonstrates differences across the anatomical references. These differences align with previous studies reporting greater accuracy and predictability for dental arch expansion when cusps or occlusal surfaces are used as references, in contrast with gingival margin landmarks [7, 17]. To account for the paired nature of the data and the non-independence of repeated measurements within patients, mixed-effects Bland–Altman models were used, thus incorporating patient random intercepts to partition within- and between-patient variability. This approach strengthens the validity of the agreement estimates, despite limitations of Bland–Altman testing when applied to clustered data, as repeated measures may still introduce residual correlation and reduce generalizability.

The algorithm for selecting reference points and centers of rotation utilized by Invisalign remains unknown, which represents a major limitation of the present study, as the internal assumptions and computational procedures cannot be independently verified. However, the near-zero bias and minimal dispersion observed in measurements using occlusal and cusp references stand in contrast to the systematic bias and wider LoA found at the gingival margin. This discrepancy suggests that the Invisalign measurement output is more closely aligned with hard tissue references (cusp tips and occlusal points), rather than the less reliable gingival margin, although this conclusion should be interpreted cautiously. Previous studies have similarly noted greater reliability of occlusal and cusp references compared with gingival margins in long-term post-orthodontic and relapse studies [29], but further independent research is required to confirm these findings.

Dental arch expansion validation using occlusal and cusp references is consistent with the conclusion of the previous validation study on overbite and overjet: [10] there was no significant fixed bias. Both results suggest that the two measurement systems (Geomagic and ClinCheck) are not systematically different when using stable anatomical references. A methodological strength of our investigation is the expanded scope of validation beyond the initial treatment plan. The study on overjet and overbite validated only the difference between the baseline model and the planned outcome (pretreatment vs. predicted), both at the first ClinCheck. The present study incorporated the observed expansion (derived from the initial stage of the first refinement) in addition to the predicted expansion. By validating data across 2 distinct ClinChecks —comparing the pretreatment stage to both the predicted and observed outcomes—the findings of the present study offer a robust assessment of the longitudinal consistency and reliability of ClinCheck expansion measurements across multiple records of each patient. Particularly, the observed expansion corresponded to the treatment stage prior to the first refinement, and was included to assess the consistency of measurement reliability across multiple ClinCheck records.

The discrepancy (observed expansion minus predicted expansion) yielded the most consistent results across all measurement references. This variable isolates the true clinical execution error or the unaccounted biological response with greater fidelity than either the predicted or observed expansion alone. It is less sensitive to baseline variability because it compares two outputs derived from the same reference system rather than relying on absolute values. By focusing on the difference between predicted and observed expansion, the discrepancy reduces the influence of anatomical reference definition or segmentation inconsistencies that can affect baseline measurements. The high consistency observed in the discrepancy offers a robust metric for quantifying treatment efficacy, while its tight LoA validates the interchangeability of the Geomagic measurement system and ClinCheck data when assessing how much the patient’s actual progress deviates from the plan. For future metrology studies, discrepancy may serve as a practical benchmark for assessing the reliability of digital treatment planning tools, as it is inherently less affected by baseline measurement error than either predicted or observed expansion alone.

Several limitations must be acknowledged. First, data was retrieved by a single operator, which may limit reproducibility. Second, patient selection was retrospective, and repeated measurements within patients were not independent, although clustering was addressed statistically. Third, comparative analysis was indirect, as ClinCheck relies on a proprietary non released algorithm, and use of first-refinement models served to assess the consistency of measurement reliability across multiple ClinCheck records rather than providing a direct validation of clinical outcomes. Fourth, the generalizability of our findings is restricted to adult patients treated with SmartTrack by a single clinician. Fifth, all STL files were obtained directly from the ClinCheck interface, including the predicted outcome model, which does not exist as a raw pre-segmentation file. We did not compare the original impression STL files submitted to Align Technology with the segmented ClinCheck models. Future studies should investigate potential discrepancies introduced during the segmentation process. Finally, the gingival margin in ClinCheck STL files is digitally processed, which limits the accuracy of the reference plane. Therefore, following previously published protocols, we constructed the reference plane using the palatal interproximal papillae. These structures exhibit lower variability than vestibular gingival margins, which might be more affected by segmentation. They have provided stable reference points in similar measurement workflows.

Importantly, agreement does not equate to absolute accuracy. While the LoA observed ( ≤ ± 1 mm for occlusal and cusp, ≤ ±1.8 mm for gingival) are within clinically acceptable thresholds for arch width changes, they should be interpreted as measures of concordance rather than proof of predictive accuracy. Future studies incorporating longitudinal clinical outcomes and multi-operator designs are needed to confirm the robustness of these findings.

Despite the rapid global adoption of clear aligner therapy, concerns about clinical performance and reliability of measurements in digital treatment plans remain. The comparative analysis of this study is intended to serve as a critical methodological benchmark for future investigations. The presented findings can stimulate further research into accuracy, longitudinal consistency, and optimal reference selection across different digital treatment planning methodologies, thereby contributing to the evidence base that guides the clinical application of CAT.

## Conclusions

- Predicted and observed expansion, as well as their discrepancy, showed narrow ranges of bias across cusp, occlusal, and gingival references, indicating that expansion values derived from Invisalign arch width tables are broadly consistent with those obtained from direct measurements in virtual models.
- Expansion based upon occlusal and cusp references showed greater precision and agreement than gingival references, supporting the use of hard-tissue references as more stable anatomical points for metrological assessment.
- Transverse changes derived from arch width values in the Invisalign ClinCheck platform were metrologically consistent with independent measurements, although this agreement should not be interpreted as full validation of the proprietary algorithm or as proof of clinical reliability.

## Data Availability

Data can be provided under reasonable request.

## References

[1] Alfawal AMH, Burhan AS, Mahmoud G et al (2022) The impact of non-extraction orthodontic treatment on oral health-related quality of life: clear aligners versus fixed appliances-a randomized controlled trial. Eur J Orthod 44:595–602. 10.1093/ejo/cjac012 35395075 10.1093/ejo/cjac012

[2] Meade MJ, Weir T (2022) A survey of orthodontic clear aligner practices among orthodontists. Am J Orthod Dentofac Orthop 162:e302–e311. 10.1016/j.ajodo.2022.08.018 10.1016/j.ajodo.2022.08.018 36202697

[3] Meade MJ, Weir T (2023) Clear aligner therapy procedures and protocols of orthodontists in New Zealand. Australasian orthodontic J 39:123–135. 10.2478/aoj-2023-0031

[4] Meade MJ, Weir T, Seehra J, Fleming PS (2024) Clear aligner therapy practice among orthodontists in the United Kingdom and the Republic of Ireland: A cross-sectional survey of the British Orthodontic Society membership. J Orthod 51:120–129. 10.1177/14653125231204889 37830274 10.1177/14653125231204889 PMC11141077

[5] Miranda e Paulo D, Moreira-Santos LF, Tavares MC et al (2024) Clear aligner therapy practices among orthodontists practicing in Canada. Prog Orthod 25:27–28. 10.1186/s40510-024-00525-3 38972901 10.1186/s40510-024-00525-3 PMC11228011

[6] Bruni A, Serra FG, Gallo V et al (2021) The 50 most-cited articles on clear aligner treatment: A bibliometric and visualized analysis. Am J Orthod Dentofac Orthop 159:e343–e362. 10.1016/j.ajodo.2020.11.029 10.1016/j.ajodo.2020.11.029 33653640

[7] Tien R, Patel V, Chen T et al (2023) The predictability of expansion with Invisalign: A retrospective cohort study. Am J Orthod Dentofac Orthop 163:47–53 10.1016/j.ajodo.2021.07.032 10.1016/j.ajodo.2021.07.032 36195544

[8] Shailendran A, Weir T, Freer E, Kerr B (2022) Accuracy and reliability of tooth widths and Bolton ratios measured by ClinCheck Pro. Am J Orthod Dentofac Orthop 161:65–73. 10.1016/j.ajodo.2020.06.048 10.1016/j.ajodo.2020.06.048 34417034

[9] Smith JM, Weir T, Kaang A, Farella M (2022) Predictability of lower incisor tip using clear aligner therapy. Prog Orthod 23:37–12. 10.1186/s40510-022-00433-4 36336726 10.1186/s40510-022-00433-4 PMC9637687

[10] Meade MJ, Blundell H, Weir T (2024) Predicted overbite and overjet changes with the Invisalign appliance: a validation study. Angle Orthod 94:10–16. 10.2319/041323-269.1 37655807 10.2319/041323-269.1 PMC10928931

[11] de-la-Rosa-Gay C, Valmaseda-Castellón E, Figueiredo R, Camps-Font O (2025) Factors affecting expansion predictability of clear aligner treatment. Clin Oral Investig 29:257. 10.1007/s00784-025-06328-y 40257582 10.1007/s00784-025-06328-y PMC12011961

[12] Zhou N, Guo J (2020) Efficiency of upper arch expansion with the Invisalign system. Angle Orthod 90:23–30. 10.2319/022719-151.1 31368778 10.2319/022719-151.1 PMC8087062

[13] Lione R, Paoloni V, Bartolommei L et al (2021) Maxillary arch development with Invisalign system: Analysis of expansion dental movements on digital dental casts. Angle Orthod 91:433–440. 10.2319/080520-687.1 33570617 10.2319/080520-687.1 PMC8259755

[14] Bouchant M, Saade A, El Helou M (2023) Is maxillary arch expansion with Invisalign^®^ efficient and predictable? A systematic review. Int Orthod 21. 10.1016/j.ortho.2023.100750 10.1016/j.ortho.2023.100750 36989750

[15] Vidal-Bernárdez M, Vilches-Arenas Á, Sonnemberg B et al (2021) Efficacy and predictability of maxillary and mandibular expansion with the Invisalign^®^ system. J Clin Exp Dent 13:e669–e677. 10.4317/jced.58315 34306530 10.4317/jced.58315 PMC8291161

[16] Vidal Bernárdez ML, Vilches Arenas Á, Sonnemberg B et al (2022) EX30 vs. SmartTrack materials in maxillary expansion with the Invisalign system. J Clin Orthod 56:343–350 35948311

[17] Houle J-P, Piedade L, Todescan J, Pinheiro FHSL (2017) The predictability of transverse changes with Invisalign. Angle Orthod 87:19–24. 10.2319/122115-875.1 27304231 10.2319/122115-875.1 PMC8388591

[18] Solano-Mendoza B, Sonnemberg B, Solano-Reina E, Iglesias-Linares A (2017) How effective is the Invisalign^®^ system in expansion movement with Ex30′ aligners? Clin Oral Investig 21:1475–1484. 10.1007/s00784-016-1908-y 27435982 10.1007/s00784-016-1908-y

[19] Albarki Z, Weir T, Meade MJ (2025) Efficacy of planned root torque changes of instanding maxillary lateral incisors with the Invisalign appliance. Am J Orthod Dentofac Orthop 168:367–378. 10.1016/j.ajodo.2025.04.016 10.1016/j.ajodo.2025.04.016 40358566

[20] Blundell HL, Weir T, Byrne G (2022) Predictability of overbite control with the Invisalign appliance comparing SmartTrack with precision bite ramps to EX30. Am J Orthod Dentofac Orthop 162:e71–e81. 10.1016/j.ajodo.2022.05.012 10.1016/j.ajodo.2022.05.012 35750579

[21] Blundell HL, Weir T, Byrne G (2023) Predictability of anterior open bite treatment with Invisalign. Am J Orthod Dentofac Orthop 164:674–681. 10.1016/j.ajodo.2023.04.017 10.1016/j.ajodo.2023.04.017 37330726

[22] Rajan N, Weir T, Meade MJ (2025) Efficacy of planned moderate to severe torque changes in mandibular central incisors with an initial series of Invisalign aligners: a retrospective cohort study. Angle Orthod 95:12–18. 10.2319/061724-473.1 39313216 10.2319/061724-473.1 PMC11662367

[23] Rajan N, Weir T, Meade MJ (2024) Efficacy of planned moderate to severe torque changes in maxillary central incisors with the Invisalign appliance: A retrospective investigation. Am J Orthod Dentofac Orthop 166:375–383. 10.1016/j.ajodo.2024.06.008 10.1016/j.ajodo.2024.06.008 39046382

[24] Maree A, Kerr B, Weir T, Freer E (2022) Clinical expression of programmed rotation and uprighting of bilateral winged maxillary central incisors with the Invisalign appliance: A retrospective study. Am J Orthod Dentofac Orthop 161:74–83. 10.1016/j.ajodo.2020.06.049 10.1016/j.ajodo.2020.06.049 34563426

[25] Sousa MVS, Vasconcelos EC, Janson G et al (2012) Accuracy and reproducibility of 3-dimensional digital model measurements. Am J Orthod Dentofac Orthop 142:269–273. 10.1016/j.ajodo.2011.12.028 10.1016/j.ajodo.2011.12.028 22858338

[26] von Elm E, Altman DG, Egger M et al (2007) The Strengthening the Reporting of Observational Studies in Epidemiology (STROBE) Statement: Guidelines for Reporting Observational Studies. Epidemiology 18:800–804. 10.1097/EDE.0b013e3181577654 18049194 10.1097/EDE.0b013e3181577654

[27] Halonen JI, Erhola M, Furman E et al (2020) The Helsinki Declaration 2020: Europe that protects. Lancet Planet Health 4:e503–e505. 10.1016/S2542-5196(20)30242-4 33159874 10.1016/S2542-5196(20)30242-4

[28] Blundell HL, Weir T, Kerr B, Freer E (2021) Predictability of overbite control with the Invisalign appliance. Am J Orthod Dentofac Orthop 160:725–731. 10.1016/j.ajodo.2020.06.042 10.1016/j.ajodo.2020.06.042 34373153

[29] Forde K, Storey M, Littlewood SJ et al (2018) Bonded versus vacuum-formed retainers: a randomized controlled trial. Part 1: stability, retainer survival, and patient satisfaction outcomes after 12 months. Eur J Orthod 40:387–398. 10.1093/EJO/CJX058 29059289 10.1093/ejo/cjx058

[30] Donatelli RE, Lee S-J (2013) How to report reliability in orthodontic research: Part 1. Am J Orthod Dentofac Orthop 144:156–161. 10.1016/j.ajodo.2013.03.014 10.1016/j.ajodo.2013.03.014 23810057

[31] Donatelli RE, Park J-A, Alghamdi YMA et al (2022) Assessment of reliability in orthodontic literature: A meta-epidemiological study. Angle Orthod 92:409–414. 10.2319/081021-625.1 35099528 10.2319/081021-625.1 PMC9020402

